# Dobutamine in Paediatric Population: A Systematic Review in Juvenile Animal Models

**DOI:** 10.1371/journal.pone.0095644

**Published:** 2014-04-22

**Authors:** Victoria Mielgo, Adolf Valls i Soler, Carmen Rey-Santano

**Affiliations:** 1 Experimental Research Unit, Biocruces Health Research Institute, Cruces University Hospital, Barakaldo, Spain; 2 Department of Pediatrics, School of Medicine and Dentistry, University of the Basque Country, Barakaldo, Spain; Nottingham University, United Kingdom

## Abstract

**Objective:**

Although dobutamine is widely used in neonatal clinical practice, the evidence for its use in this specific population is not clear. We conducted a systematic review of the use of dobutamine in juvenile animals to determine whether the evidence from juvenile animal experiments with dobutamine supported the design of clinical trials in neonatal/paediatric population.

**Methods:**

Studies were identified by searching MEDLINE (1946–2012) and EMBASE (1974–2012). Articles retrieved were independently reviewed by three authors and only those concerning efficacy and safety of the drug in juvenile animals were included. Only original articles published in English and Spanish were included.

**Results:**

Following our literature search, 265 articles were retrieved and 24 studies were included in the review: 17 focused on neonatal models and 7 on young animal models. Although the aims and design of these studies, as well as the doses and ages analysed, were quite heterogeneous, the majority of authors agree that dobutamine infusion improves cardiac output in a dose dependent manner. Moreover, the cardiovascular effects of dobutamine are influenced by postnatal age, as well as by the dose used and the duration of the therapy. There is inadequate information about the effects of dobutamine on cerebral perfusion to draw conclusions.

**Conclusion:**

There is enough preclinical evidence to ensure that dobutamine improves cardiac output, however to better understand its effects in peripheral organs, such as the brain, more specific and well designed studies are required to provide additional data to support the design of clinical trials in a paediatric population.

## Introduction

The synthetic catecholamine dobutamine is a relatively cardioselective agent with significant cardiac α1- and β1/β2-adrenoreceptor-mediated direct inotropic effects and limited chronotropic action [1]. Cardiovascular compromise is a frequently condition in critically ill preterm and term infants which contributes to morbidity and mortality in these population. In the diagnosis and treatment of this pathology blood pressure, blood flow and cardiac output are some of the most determinant parameters [2]. The use of inotropes is common in neonates with cardiovascular compromise, being dobutamine introduced for the management of neonatal cardiovascular compromise two decades ago [1].

Dobutamine and dopamine are the inotropes most commonly used in neonatal intensive care; however, as many medicines used in children, especially neonates, have never been tested to the level seen in adult healthcare, the dose is established by simple extrapolation from adults without taking into account age-related differences.

In order to increase availability of medicines authorised for children as well as to increase the information available on the use of medicinal products in the paediatric population, the EU established a regulation on medicinal products for paediatric use (Regulation (EC) No 1901/2006) [3]. Notably, in the European Medicines Agency priority list (EMA/480197/2010) for studies into off-patent paediatric medicinal products published in 2010, dobutamine appeared as a drug of interest [4].

Nonetheless given the difficulties of conducting pharmacological research in children and more specifically in the neonatal population, the performance of experimental studies in suitable animal species is a common practice. Preclinical findings, and especially juvenile animal data, can be useful to generate information to support the use of a specific drug in paediatric populations. Specifically, it is important that unlike models in adult animals, those based on juvenile and in particular neonatal animals allow for the fact that drug concentration (PK) and drug response (PD) may be different in immature and developing organs. In particular in this case, it is important to focus the review on juvenile and in particular neonatal animals because there are a number of developmental variations of structure and function of the cardiovascular system that suggest a different response to the drug.

The aim of this study was to conduct a systematic review on the use of dobutamine in juvenile animal models and to summarise and assess where there is enough conclusive preclinical evidence to guide a neonatal/paediatric clinical trial to support its use in this population.

## Methods

### Identification of Studies

The literature search was restricted to published results of animal studies and the effects of dobutamine. An electronic search was conducted using MEDLINE (from 1946 to July 2012), and EMBASE (from 1980 to July 2012) with the following combinations of terms: “dobutamine AND different animal models (using the following strategy: pig* OR swine OR calf OR lamb OR rat OR mouse OR mice OR foal OR horse OR animal*) AND juvenile animals (using the following strategy: juvenile OR infant OR fetal OR foetal OR neonatal OR newborn OR preterm OR pup OR term OR near-term)”.

The dobutamine heading included different related terms such as toxicity, pharmacology, pharmacokinetics, drug administration, adverse reactions, drug concentration, therapy, as well as various different brand and generic drug names.

### Inclusion Criteria and Data Extraction

During screening, only studies reported in English or Spanish, and conducted in an “in vivo” juvenile/neonatal animal model, were included. Conference papers, book chapters and review papers were excluded, as were studies focused on adult animal models. In addition, studies were included only if the study assessed the effect of dobutamine in juvenile animal models and dobutamine was not tested in combination with other treatments.

After excluding duplicates, titles and abstracts were screened by two authors to assess whether they were relevant for this review and should be subjected to further evaluation; any disagreements were resolved by discussion and if it was not possible to reach a consensus a third author determined eligibility. Finally, the full text of articles considered relevant was evaluated by the three authors.

Relevant information such as animal model and species, age and number of animals, dose and duration of dobutamine therapy, comparison groups and outcomes, was extracted from each study entered into an electronic database and analysed. To assess the quality of the studies the following parameters were taken into account: a) randomisation of the intervention; b) blinding of the intervention; c) comparison to a control group and d) statement of compliance with animal welfare regulations. The heterogeneity of the studies included in the review precluded meta-analysis.

## Results

### Description of the Studies

Although the research was focused on articles in Spanish and English, none of the article retrieved was written in Spanish, so all the articles included in the review were in English.

**Figure 1:**
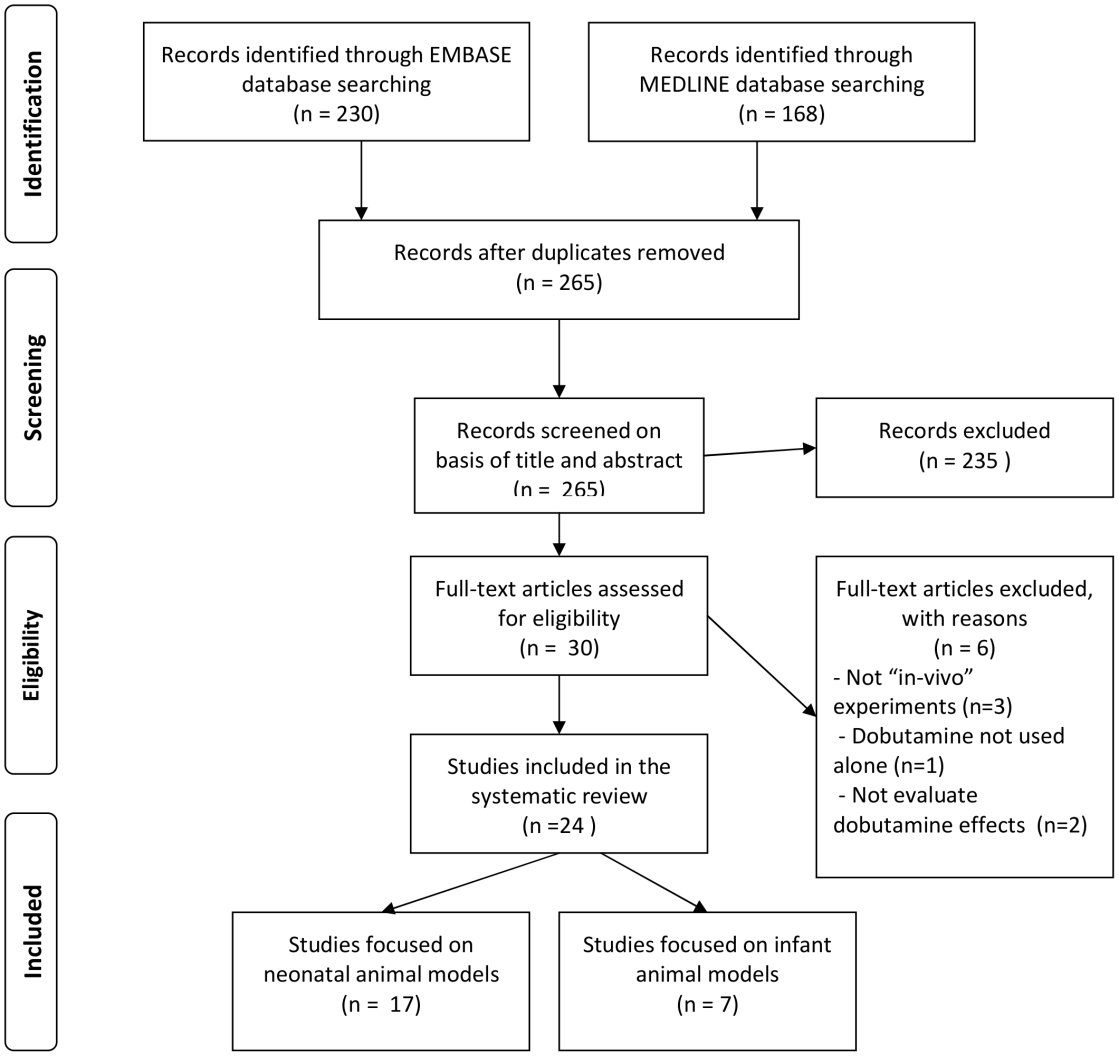
Flow chart of study selection. Flow chart showing the results of the search and reasons for exclusion of studies for systematic review.

A total of 265 articles were retrieved from the electronic search. After application of the inclusion criteria, only 24 studies were included in the systematic review; 17 focused on neonatal models and 7 on young animal models. The corresponding flow diagram is shown in Figure 1.

The characteristics of the studies focused on neonatal animal models are described in Table 1. Only one study focused on preterm animals. In the 17 articles in neonatal models included in the review, nine were performed in piglets, four in lambs, three in puppies and one in foals. A variety of animal models were used including: four studies in hypoxic animals, seven in healthy animals, two studies comparing hypoxic and normoxic conditions and the other four considering different specific conditions (pulmonary hypertension; endotoxic shock; hypotension and hypoplastic left heart syndrome). Moreover, 5 studies focused only on the effects of dobutamine whereas the other 12 studied the effects of different drugs and compared them with those of dobutamine: as a comparison drug, dopamine was used in ten studies; epinephrine in four; isoproterenol in four; norepinephrine in two; milrinone in two; nitroprusside in one and vasopressin in one. The dose range used in the studies varied from 0.5 to 80 µg/kg/min. The duration of the treatment and the infusion pattern also varied between the studies.

**Table 1:** Characteristics of neonatal animal studies included in the systematic review.

STUDY	ANIMAL MODEL	DOBUTAMINE DOSES	NUMBER OFANIMALS	COMPARISON GROUPS	DOBUTAMINE EFFECTS
Nachar R.A.et al. 2011 [5]	Newborn piglets(10±3 day old;2.4±0.6 kg)	5, 10, 15, 20, 25 and30 µg/kg/min for15 min	N = 7/DOP, DOB and epinephrinegroups; N = 6/milrinone group; N = 4/norepinephrine group	DOP: 5–30 µg/kg/min;epinephrine:0.25–2 µg/kg/min;norepinephrine:0.25–1.5 µg/kg/min; milrinone:50 µg/kg +0.375and 0.75 µg/kg/min.	HR increase in a dose-dependent manner. Low tomoderate doses increase MABP and systemic andregional BF. At 5 and 10 µg/kg/min brain, renaland intestinal BP and O_2_ saturation increase.Carotid BF and BP increase at 10 µg/kg/min.
Joynt C.et al. 2010 [6]	Hypoxic newbornpiglets (1–3 days;1.5–2.3 kg)	20 µg/kg/min for 2h	N = 6/group N = 4/sham group	Sham group; saline(hypoxic group);EP: 0.5 µg/kg/min;milrinone:0.75 µg/kg/min	CO and SV improve without differences in SVR.MABP, carotid and intestinal BF and DO_2_ increasewithout changes in renal perfusion. There are nodifferences in troponin I, lactate and histologicalfeatures of ischaemia.
Al-Salam Z.et al. 2008 [7]	Hypoxic newbornpiglets (1–3 days;1.5–2.3 kg)	5, 10 or 20 µg/kg/minfor 2h	N = 8/group N = 6/sham group	Sham group; saline(hypoxic group)	There are no differences between groups in HR,MABP. At 20 µg/kg/min plasma thromboxaneB2 increases from baseline and a platelet aggregationdysfunction and decrease in platelet number are observed.
Al-Salam Z. et al. 2007 [8]	Hypoxic newbornpiglets (1–3 days;1.5–2.3 kg)	5, 10 or 20 µg/kg/minfor 2h	N = 8/group N = 6/sham group	Sham group; saline(hypoxic group)	At 20 µg/kg/min CO and SV increase, with amoderate effect at 5 and 10 µg/kg/min, withoutchanges in HR, MABP and SVR. The PVR decreaseswith a modest increase in PAP. At 20 µg/kg/minthere is a transient improvement in mesentericperfusion, with no effect on the carotid and renalBF or DO_2_
Barrington K.J.2001 [19]	Normoxic and hypoxicnewborn piglets(1–3 days)			DOP; epinephrine	CO increases but BP only increases at very highdoses, renal and bowel perfusion are unaffectedby short term infusion, but both increase duringmore prolonged treatment
Cheung P-Y.et al. 1999 [9]	Newborn piglets(1–3 days;1.2- 2.2 kg)	5, 10, 20 and 50 µg/kg/min for 15 min(randomly given), with15 min rests betweendoses+a infusion at10 µg/kg/min for 2 h	N = 13	No	Short infusion: CO and HR increase in adose-dependent manner, without changes inSV. MABP modestly increases at 50 µg/kg/min.At 20 and 50 µg/kg/min PAP increases and SVRdecreases with no changes in PVR. Mesenteric andrenal BF are not affected. Prolonged infusion: COand SV increase with a transient tachycardia. MABPis not altered and PAP increase at 60 min butdecrease towards baseline at the end. SVR andPVR decrease. Mesenteric and renal BF increaseafter 60 min, with a decrease in VR.
Riordan C.J.et al. 1996 [10]	Newborn pigletshypoplastic Left HeartSyndrome (1- 2 weeks)	5 and 15 µg/kg/min forat least 10 min	N = 6	DOP: 5 and15 µg/kg/min;Epinephrine: 0.05 and0.1 µg/kg/min	At 15 µg/kg/min the Q_p_/Q_s_ ratio increases andDO_2_ decreases. At increasing doses the A-V O_2_significantly increases. CO increases.
Ferrara J.J.et al. 1995 [11]	Term (1–14 days)and premature(90% of termgestation) piglets	Incremental doses: 5, 10and 15 µg/kg/min, with20 min rests between doses	Preterm (n = 16); 1–2 day old(n = 18); 10–14 day old (n = 16)	DOP: 5, 10 and15 µg/kg/min	Preterm animals: HR increases moderately in adose-dependent manner, without changes inCO, MABP or BF to the studied organs.Term animals: MABP increases at 15 µg/kg/minand CO at the highest dose. HR, brain and heart BFincrease in a dose-dependent manner, without changesin renal BF and a small decrease in intestinal BF
Nudel D.B.et al. 1991 [12]	Hypoxic piglets(2–4 days and13–17 days)	Sequential 10 mininfusions of 2, 5 and15 µg/kg/min	2–4 days (n = 21) 13–17days (n = 27)	Sequential 10 min infusionsof 2, 5 and 15 µg/kg/min ofDOP or 0.05, 0.13 and0.39 ml/min of saline	Normoxaemia: HR and CO increase in both age groupsand total artery and renal resistances decrease in theyoungest. Hypoxaemia: Hypoxaemia reducesHR and contractility response to DOB in olderpiglets with little or no effect on CO and totalarterial, mesenteric and carotid resistance.
Penny D.et al. 2001 [23]	Lambs (1–2 days,7–10 days and6–8 weeks)	Incremental doses:1–40 µg/kg/min	1–2 days (n = 7); 7–10 days(n = 7); 6–8 weeks (n = 8)	No	In all groups DO_2_, HR and CO increase and MABPdecreases. VO_2_ is higher at 1–2 days old. Pulmonaryartery O_2_ content decreases in 1–2 day old andincreases in 7–8 day old and 6–8 week old animals.At 1–2 days old the temperature increases withno changes in the other groups
Smolich J.J.et al. 2000 [24]	Lambs (1–2 days,7–10 days and6–8 weeks)	Incremental doses:0.5–40 µg/kg/min	1–2 days (n = 6); 7–10 days(n = 7); 6–8 weeks (n = 6)	No	PAP increases at 1–2 and 7–10 days old, and decreasesat 6–8 weeks old. In all groups there is no change inMABP with a dose-dependent increase in CO anddecreases PVR and SVR.
Crowley M.R.et al. 1991 [20]	Newborn lambs withpulmonaryhypertension(3–5 days)	Incremental doses:5–20 µg/kg/min	N = 10	Isoproterenol:0.05–1 µg/kg/min; DOP: 3–30 µg/kg/min;Nitroprusside: 0.5–10 µg/kg/min	PAP decrease at 5 and 10 µg/kg/min, with no changesat higher doses. PVR decrease. CO increase withincreasing doses. At higher dose SV increase andSVR decrease. At 15 and 20 µg/kg/min HR increase.There are no changes in MABP.
ÓLaughlin M.P.et al. 1987 [21]	UnanaesthetizedHypoxemic NewbornLambs	10, 20, 40 and80 µg/kg/min	N = 15	Isoproterenol: 0.1, 0.4, 0.7 and1 µg/kg/min; DOP: 10, 20, 40and 80 µg/kg/min	CO and HR increase with all the dosages tested.SVR decreases between 20–80 µg/kg/min.
Goto M. et al.1991 [22]	Newborn puppiesendotoxic shock(2–10 days)	5 µg/kg/min	N = 14/LPS group; N = 9/DOPand DOB groups; N = 11/DOP+IND and IND groups;N = 8/DOB+IND group	LPS group; DOP(15 µg/kg/min);IND (1.5 mg/kg)	HR is unchanged and SVR is maintained at thehigh level. MABP is stable for the first 60 minand then declines. The hypotension and COdecreases are attenuated when compared toLPS group.
Driscoll D.J.et al. 1980 [28]	Newborn puppies(0–10 days) andadults	Incremental doses: 2 to 50 µg/kg/min	N = 11 puppies;N = 5 adults	Isoproterenol: 0.05 to1.25 µg/kg/min; DOP: 2 to50 µg/kg/min	Adults: MABP, HR, CO and renal BF increase.Puppies: HR increases without changes in MABP,CO and renal BF.
Driscoll D.J.et al. 1979 [25]	Puppies(From 3 to 65 days)	Incremental doses:2 to 50 µg/kg/min	N = 24	Isoproterenol: 0.05 to1.25 µg/kg/min; DOP: 2 to50 µg/kg/min	At 20 µg/kg/min MABP and HR increase.At 50 µg/kg/min CO increases. There areno changes in SVR and renal artery BF with anincrease in renal VR at 20 and 50 µg/kg/min
Valverde A.et al. 2006 [27]	Hypotensive newbornfoals (1–5 days)	4 and 8 µg/kg/minfor 15 min.	N = 6	Norepinephrine: 0.3 and1 µg/kg/min; Vasopressin:0.3 and 1 mU/kg/min	CO, MABP and DO_2_ increase and VO_2_ and O_2_extraction decrease. SVR decreases at highinfusion rates

***Abbreviations:*** DOP: dopamine; LPS: lipopolysaccharide; IND: indomethacin; CO: cardiac output; HR: heart rate; SV: stroke volume; MABP: mean arterial blood pressure; (S)VR: (systemic) vascular resistance; BF: blood flow; VO_2_: O_2_ consumption; DO_2_: O_2_ delivery; PVR: pulmonary vascular resistance; PAP: pulmonary artery pressure.

Table 2 summarises the characteristics of the seven studies focused on juvenile animals (infants or adolescents): six of them were in piglets and one in healthy foals. The animal models differ, using healthy animals in four studies and disease models in the other three. In five of them the effect of dobutamine was compared with that of other drugs (namely, dopamine in four studies; epinephrine in two; isoproterenol in one; norepinephrine in two; milrinone in two and phenylephrine in one), while just two articles focused on the effects of dobutamine in a model of post-resuscitation left ventricular dysfunction. The dose ranged from 2 to 32 µg/kg/min.

**Table 2:** Characteristics of young animal studies included in the systematic review.

STUDY	ANIMALMODEL	DOBUTAMINEDOSES	NUMBER OFANIMALS	COMPARISONGROUPS	DOBUTAMINE EFFECTS
Vasquez A.et al. 2004 [13]	Young pigs (24±0.4 kg)post-resuscitation leftventricular dysfunction	2, 5 and 7.5 µg/kg/min for 6 h	N = 20	Control group (placebo)	Ventricular systolic and diastolic function improveswithin minutes of infusion start and persists at 6 hfor the 5 and 7.5 µg/kg/min. HR and CO increasewith all doses, but only affect myocardial VO_2_ at7.5 µg/kg/min.
McGovern J.J.et al. 2000 [14]	Young pigs rightventricular injury(9–12 kg)	10 µg/kg/min for20 min	N = 10	DOP at 10 µg/kg/min;epinephrine at0.1 µg/kg/min	Pulmonary BF increases and PVR decreases withoutchanges in PAP. Input resistance, right ventriclecontractility and increased total hydraulic powerdecrease, without changes in transpulmonaryvascular efficiency
Kern K.B.et al. 1997 [15]	Juvenile pigs (29±1 kg)postresuscitation leftventricular dysfunction	5 or 10 µg/kg/min	10 µg/kg/min(n = 14); 5 µg/kg/min (n = 5);controls (n = 8)	Control group	Pulmonary capillary wedge pressure, left ventricularend-diastolic pressure and time constant of leftventricular isovolumic relaxation decrease.HR and left ventricular ejection fraction increase
Cassidy S.C.et al. 1997 [16]	Pigs (3 weeks old)	20 µg/kg/min	N = 9	Epinephrine andnorepinephrine: 1.5 µg/kg/min; DOP: 12 µg/kg/min; isoproterenol: 0.5 µg/kg/min and phenylephrine:20 µg/kg +2 µg/kg/min	HR, CO, dP/dt max and preload recruitable strokework increase, without changes in SVR, end-systolicelastance, dP/dt min and left ventricularchamber stiffness
Tighe D.et al. 1995 [17]	Adolescent pigs withsepsis (25–30 kg)	10 µg/kg/min for 6h	N = 25	Sham group; control group;dopexamine: 10 µg/kg/min;colloid - hydroxyethylstarch group	CO and SV increase and SVR decreases withoutchanges in HR, MABP, VO2, DO_2_ and O_2_ extractionratio in either whole-body or liver and splanchnic level.Dobutamine causes considerable deterioration of hepaticultrastructure compared to other groups
Fisher D.H.et al. 1988 [18]	Healthy conscious pigs(1–2 months old)	Incremental dose:2 to 32 µg/kg/minfor 30 min each dose.	N = 12	DOP: 2 to 32 µg/kg/min	At doses >16 µg/kg/min MABP and SVR decreaseand CO and HR with no changes in SV. Renal VRdecreases with doses in the range of 16–32 µg/kg/minassociated with an increase in renal BF. There were nochanges PAP, PVR or left atrial pressure
Craig C.A.et al. 2007 [26]	Healthy foals(1–2 weeks old)	2.5, 5 and 10 µg/kg/min for a minimum of15 min	N = 7	Norepinephrine: 0.05, 0.1,0.2 and 0.4 µg/kg/min	HR, SV, CO, DO_2_, VO_2_ and ventricular stroke workincrease; MABP and PAP increase slightly whilepreload pressure changes were variable. SVR, PVRand FTOE decrease

***Abbreviations:*** DOP: dopamine; DOB: dobutamine; HR: heart rate; MABP: mean arterial blood pressure; CO: cardiac output; BF: blood flow; (S)VR: (systemic) vascular resistance; PAP: pulmonary artery pressure; PVR: pulmonary vascular resistance; SV: stroke volume; DO2: O2 delivery; VO2: O2 consumption; LV dp/dt: first derivative of left ventricle pressure with respect to time.

In general, although studies were conducted in different animal species, more than 62% of them were performed in pigs [5]–[19].

Only one of the studies included was blinded [6], while randomization was reported in 42% of the studies [6]–[9], [11], [13], [14], [16], [17], [20]. Similarly, 42% of the studies used a control group [5]–[8], [12], [13], [15], [17], [21], [22]. Some studies assessed the effects of dobutamine by comparison with baseline data and/or the effect of other drugs. Also, a 21% of the studies did not report any compliance with animal welfare regulations.

### Cardiovascular Effects

In general, it seems that the cardiovascular effects of dobutamine are influenced by postnatal age, as well as by the dose and duration of the treatment. Although the design and aims of the studies are relatively heterogeneous, as were the doses used and the conditions, 75% of the studies report that dobutamine infusion improves cardiac output in a dose dependent-manner [6], [8]–[13], [16]–[21], [23]–[27].

Some found no changes in arterial blood pressure [7]–[9], [17], [20], [24], [28], but in a few studies the arterial blood pressure increased at the highest dose studied [6], [11], [19], [26], [27]. Similarly, some of the studies showed an increase in heart rate in a dose-dependent manner [5], [9], [11]–[13], [16], [18], [23], [26], [28]. An increase in stroke volume was mainly observed in studies using prolonged infusion (from 2 to 6 h) at doses higher than 10 µg/kg/min [6], [8], [9], [17].

In an effort to detect any patterns in the results we looked at the studies performed in pig, since it is the specie more frequently used. In this regard, most authors agree that dobutamine infusion improves cardiac output mainly at doses higher than 10 µg/kg/min [6], [8]–[14], [16]–[19]. It seems that at short term infusion this improvement was mainly due to an increase in heart rate [9], [11], [12], [16], [18], whereas at long term the increment in stroke volume is more prevalent [6], [8], [9], [17].

Further, it seems that the inotropic effect of dobutamine is present both during normoxia and hypoxia, but sometimes in a more attenuated way in the latter condition [12]. Only one study focused on preterm animals and in this case an increase in heart rate was observed without changes in mean arterial blood pressure or cardiac output at any of the doses used [11].

### Other Organ Effects

Some studies evaluated the effects of dobutamine on other organs, mainly at renal, mesenteric and pulmonary levels, with results varying as a function of dose and duration of treatment.

There are 5 studies, all in neonatal animal models, on the effects of dobutamine on cerebral perfusion, which is mainly assessed by measurement of carotid blood flow. In this regard, two studies showed an increase in carotid blood flow at dose higher than 10 µg/kg/min, independent of the duration of the treatment [5], [6] and in one study that measured cerebral blood flow through radiolabeled microspheres, a dose-dependent increase (5–15 µg/kg/min) was observed [11]. However, in another study there were no changes in carotid blood flow regardless of the dose used (5–20 µg/kg/min) [8], and other authors noted an increase in carotid vascular resistance at lowest doses (2 and 5 µg/kg/min) [12].

Some authors studied carotid oxygenation by various methods: one study found no changes at any given doses (5–20 µg/kg/min) [8], while another showed an increase at 5 and 10 µg/kg/min [12]. No follow-up studies were identified on neurodevelopment in animals treated with dobutamine.

At the renal level, two studies concluded that the renal response to dobutamine depends on maturity [12], [28]. With respect to treatment duration, two studies in newborn piglets observed an increase in renal artery blood flow after 60 min of dobutamine infusion with no changes at shorter infusion times [9], [19]. In most studies there were no changes in renal blood flow after dobutamine administration [6], [8], [11], [25], but some authors studying juvenile and adult animals observed increases in renal blood flow and decreases in renal vascular resistance, especially when the drug was infused continuously [12], [18], [28].

At the pulmonary level, some studies observed a decrease in pulmonary vascular resistance [6], [8], [9], [24], [26]. In contrast, however, one study in juvenile animals reported no changes in either pulmonary vascular resistance or pulmonary artery pressure [18].

Considering models of specific diseases, the effects of dobutamine are mixed, depending on the nature of the disease. For example, in a pulmonary hypertension model, dobutamine decreased the pulmonary artery pressure and pulmonary vascular resistance at 5 and 10 µg/kg/min [20]. In a neonatal piglet model of hypoplastic left heart syndrome an increase in the Qp/Qs ratio was observed, possibly due to differential effects on systemic and pulmonary vascular resistance [10], and in a model of right ventricular injury in young swine indices of pulmonary vascular impedance were found to decrease [14].

In regard to perfusion to other organs, it should be noted that an increase in mesenteric blood flow was observed with continuous dobutamine infusion over 2 hours at doses higher than 10 µg/kg/min [6], [8], [9], [19], though there were no changes at short infusion times (10–15 min.) [9], [12], [19]. Moreover, in a study in newborn piglets an increase in intestinal and renal oxygen saturation was observed at 5 and 10 µg/kg/min [5], whereas in a study in adolescent pigs with sepsis a deterioration in liver function was observed at 10 µg/kg/min [17]. More specifically, one study described platelet aggregation dysfunction in hypoxic-reoxygenated newborn piglets treated with 10 and 20 µg/kg/min of dobutamine [7].

Only one study evaluated the use of dobutamine in premature animals (piglets born at 90% gestation) and no changes in specific organ perfusion were detected [11].

Considering the articles included in the review, none of them reported pharmacokinetics and pharmacodynamics (PK/PD) responses of dobutamine in neonatal and juvenile animals or focused on toxicity in immature systems.

## Discussion

In the present study we focused on whether the evidence available regarding the use of dobutamine in juvenile animal was sufficient and adequate to support a clinical trial and the use of this drug in paediatric/neonatal populations.

The use of well-designed neonatal animal models, to address and predict effects of dobutamine on developing organs and therefore to obtain information on the potential different safety profile from those seen in adults, are important to support a clinical trial, due to developmental variations of structure and function of the cardiovascular system. In the context of this systematic review, although there are quite a number of studies in this field in different animal species, data from term and preterm pigs are particularly relevance due to pig is a representative model for the developmental cardiovascular physiology of humans [29]–[31]. However, the marked variability in the dose used, timing and duration of treatment and study design produces discrepancies between the results of studies.

Despite the heterogeneity of the studies, most of them show that dobutamine improves cardiac output in a dose-dependent manner, both in neonatal and juvenile models. As cardiac output is a crucial parameter in neonatal cardiovascular function, its improvement may be of importance in decreasing morbidity and mortality associated with cardiovascular compromise in this population.

However, precisely the variability in these studies means that the mechanism by which this improvement is achieved is not clear. Some of the studies analysed report an increase in heart rate [5], [9], [11]–[13], [16], [18], [23], [26], [28], whereas others show an improvement in stroke volume [6], [8], [9], [17] or even an increase in mean arterial pressure [6], [11], [19], [26], [27]. Focusing on studies in pigs, as an animal model, this point seems clearer, being at short term infusion tachycardia more marked [9], [11], [16], [18], [23] and at long term infusion mainly due to an increase in stroke volume than tachycardia [6], [8], [9], [17].

The existing information about the effects on cerebral perfusion and neurodevelopmental outcomes is clearly inadequate. Although there has been some research in this area, in neonatal animal models [5], [6], [8], [11], [12], which use mainly carotid blood flow measurements to study cerebral perfusion, the results are not consistent. This is probably due to the duration of treatment, the dose regimen and the existence of differences in measurement methods (NIRS, radiolabeled microspheres, electromagnetic flow probes, ultrasonic flow probes). The variability in measurement methods used, with advantages and disadvantages between them and in many cases practical and technological limitations, make difficult to compare them and to obtain clear conclusions. Although more studies are needed to clarify the effect of dobutamine at a cerebral level to translate clear conclusions to neonatal care, some of the studies included in the review suggest a trend towards an increase in the blood flow at doses higher than 10 µg/kg/min. So in the design of future studies this point should be considered.

In addition, only one study investigated the effect of dobutamine in preterm animals [11], with insufficient power to extract reliable conclusions to predict human clinical outcomes in this particular population.

Finally, the data on adverse effects reported in some studies (such as liver damage or platelet aggregation dysfunction) related to the use of dobutamine in specific diseases [7], [17] are not conclusive. These studies should be analysed carefully, suggesting that more research is needed in this area.

### Limitations

Systematic reviews are vulnerable to various different forms of bias. Our search was limited to studies published in English referenced in electronic databases, while there may also be relevant studies that are unpublished or written in other languages. Moreover, the quality of any review is determined by that of the studies included, so any bias in studies included associated with a lack of randomization or blinded assessment could have an impact on the conclusions. In this review, the quality of included studies was overall poor with less than half of them randomly allocating animals to groups, and only one being a blinded study [6].

## Conclusions

On the one hand there is enough preclinical evidence to conclude that dobutamine improves cardiac output. On the other hand, although there has been some research on the effects of dobutamine in juvenile animal models, the heterogeneity across studies makes it difficult to obtain clear evidence to better understand its effects in peripheral organs, above all in the brain. Hence, it is necessary to perform more standardised and higher quality studies to support a clinical trial and to allow clear conclusions to be drawn concerning the rational use of dobutamine in paediatric/neonatal population.

Moreover, it is important to conduct studies in juvenile and neonatal animals designed to address specific questions of potential toxicity in developing systems, to perform PK/PD studies, at different postnatal ages, to develop evidence for individualized dosing schemes for children and also to collect data on the long-term safety of dobutamine that cannot be obtained from clinical trials.
